# The Thymic Orchestration Involving Aire, miRNAs, and Cell–Cell Interactions during the Induction of Central Tolerance

**DOI:** 10.3389/fimmu.2015.00352

**Published:** 2015-07-14

**Authors:** Geraldo Aleixo Passos, Daniella Arêas Mendes-da-Cruz, Ernna Hérida Oliveira

**Affiliations:** ^1^Molecular Immunogenetics Group, Department of Genetics, Ribeirão Preto Medical School, University of São Paulo, Ribeirão Preto, São Paulo, Brazil; ^2^Disciplines of Genetics and Molecular Biology, Department of Morphology, Physiology and Basic Pathology, School of Dentistry of Ribeirão Preto, University of São Paulo, Ribeirão Preto, São Paulo, Brazil; ^3^Laboratory on Thymus Research, Oswaldo Cruz Institute, Oswaldo Cruz Foundation, Rio de Janeiro, Rio de Janeiro, Brazil

**Keywords:** AIRE, miRNA, MTEC, thymus gland, thymocytes, cell adhesion, promiscuous gene expression, central tolerance

## Abstract

Developing thymocytes interact sequentially with two distinct structures within the thymus: the cortex and medulla. Surviving single-positive and double-positive thymocytes from the cortex migrate into the medulla, where they interact with medullary thymic epithelial cells (mTECs). These cells ectopically express a vast set of peripheral tissue antigens (PTAs), a property termed promiscuous gene expression that is associated with the presentation of PTAs by mTECs to thymocytes. Thymocyte clones that have a high affinity for PTAs are eliminated by apoptosis in a process termed negative selection, which is essential for tolerance induction. The Aire gene is an important factor that controls the expression of a large set of PTAs. In addition to PTAs, Aire also controls the expression of miRNAs in mTECs. These miRNAs are important in the organization of the thymic architecture and act as posttranscriptional controllers of PTAs. Herein, we discuss recent discoveries and highlight open questions regarding the migration and interaction of developing thymocytes with thymic stroma, the ectopic expression of PTAs by mTECs, the association between Aire and miRNAs and its effects on central tolerance.

## Introduction

The induction of central immune tolerance is an increasingly complex and intricate process that occurs within the thymus (1, 2). Inside this organ, immature thymocytes interact sequentially and in a three-dimensional architecture with two distinct structures: the cortex and the medulla. In the cortex, the double-negative (DN) and double-positive (DP) thymocytes interact with cortical thymic epithelial cells (cTECs), allowing MHC-mediated self-peptide presentation to DP thymocytes expressing the α/β T cell receptor (α/β TCR), featuring intermediate affinity/avidity. Positive selection is a result of this interaction, which causes DP thymocytes to differentiate into mature single-positive (SP) thymocytes (3, 4).

The DP thymocytes that do not undergo positive selection are eliminated through death by neglect. Thereafter, the surviving SP and DP thymocytes migrate to the thymic medulla, where they interact with medullary thymic epithelial cells (mTECs). These cells are very peculiar because they ectopically express a large set of peripheral tissue antigens (PTAs) (5–8). Therefore, it is possible to find insulin, a PTA that represents pancreatic beta cells, and a myriad of other autoantigens in the thymus.

The immunological significance of this property, which was termed promiscuous gene expression (PGE) (9–12), is associated with the presentation of PTAs by mTECs to SP and DP thymocytes. Thymocyte clones that express α/β TCR with a high affinity for PTAs are eliminated by apoptosis in a process termed negative selection or clonal deletion, which is essential for central tolerance induction (13–16). This process prevents the passage of autoreactive T cell clones to the periphery, which could provoke aggressive autoimmunity.

Therefore, the migration of thymocytes within the thymus enables the physical association of these cells with different thymic microenvironments (15, 17). Immunologists are interested in elucidating which chemotactic factors and/or adhesion molecules are involved in this process (18, 19).

Another very important factor in central tolerance is the autoimmune regulator (Aire) gene that controls the expression of a large set (but not all) of PTAs in mTECs (20, 21). Mutations in these gene that lead to a loss of Aire function can result in autoimmune polyendocrinopathycandidiasis-ectodermal dystrophy (APECED), an autoimmune disease characterized by hypoparathyroidism, candidiasis (yeast infection), and adrenal insufficiency (22–24). The mechanism of the Aire gene as a transcriptional regulator of Aire-dependent PTAs and the effect of point mutations found in the Aire gene sequence on clinical phenotypes (APECED or other autoimmune diseases) have received attention in recent years (25–27).

In addition, researchers have observed that in addition to PTAs, Aire controls the expression of microRNAs (miRNAs) in mTECs (28). In turn, miRNAs are important for the organization of thymic architecture and act as posttranscriptional controllers of PTAs (29, 30).

In this mini-review, we briefly discuss (1) the main aspects of three-dimensional thymus architecture, focusing on the migration and interaction of developing thymocytes with the thymic stroma and positive and negative selection; (2) the ectopic expression of PTAs by mTECs and role of the Aire gene; and (3) the current evidence for the link between Aire and miRNAs in thymic architecture and the induction of central tolerance.

## Thymus Architecture, Migration of Thymocytes and the Induction of Central Tolerance

**Figure 1:**
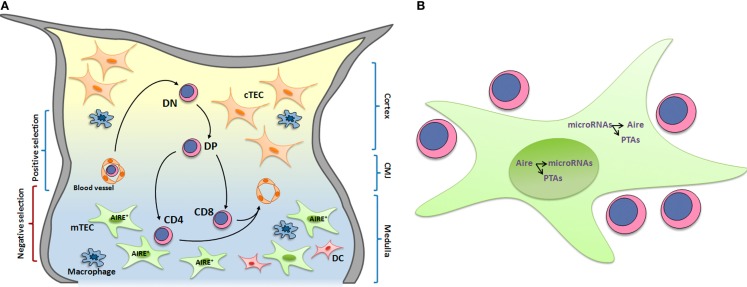
**(A)** Thymocyte development and interactions with microenvironmental cells. T cell development occurs while cells migrate and interact with thymic microenvironmental components, including thymic epithelial cells, dendritic cells, and macrophages. These interactions are responsible for the selective processes, which lead to the formation of a self-restricted T-cell repertoire. In this context, medullary thymic epithelial cells (mTECs) play an essential role by expressing AIRE, which control the expression of a set of peripheral tissue antigens (PTAs) that are presented to developing thymocytes. High avidity of MHC-PTAs presented by mTECs with TCRs lead to thymocyte death by apoptosis and consequently avoid self-reactive T cell maturation. DN, double-negative CD4^−^CD8^−^ thymocytes; DP, doublepositive CD4^+^CD8^+^; CD4, single-positive CD4^+^CD8^−^; CD8, single-positive CD8^+^CD4^−^ thymocytes; cTEC, cortical thymic epithelial cell; mTEC, medullary thymic epithelial cell. CMJ, cortico-medullary junction. **(B)** The transcriptional and posttranscriptional control pathway of promiscuous gene expression. The peripheral tissue antigens (PTAs) expressed in medullary thymic epithelial cells (mTECs) are transcriptionally controlled by Aire within the nuclear compartment, which also controls the expression of miRNAs. Within the cytoplasm, the miRNAs control Aire and PTAs.

Developing thymocytes interact with the thymic microenvironment while they migrate and differentiate within the organ. This microenvironment is subdivided into two main regions, and each region is composed of different cell types that produce soluble and non-soluble molecules that can modulate thymocyte migration and maturation (31, 32). Thymic lobules are divided into cortical and medullary regions that are connected by a cortico-medullary junction. The cortex microenvironment is filled with cTECs, thymic nurse cells (TECs-thymocyte-forming lymphoepithelial complexes), macrophages, migratory dendritic cells (DCs), and fibroblasts. The medullary region contains mTECs, macrophages, resident and migratory conventional DCs, plasmacytoid DCs, fibroblasts, and B cells (16) (Figure 1A). Both regions are filled with a network of extracellular matrix (ECM) molecules, such as type I and IV collagens, fibronectin, and laminin. Soluble molecules, such as hormones, cytokines, growth factors, chemokines, and sphingolipids, are also found in the thymus and are produced by the lymphoid and non-lymphoid compartments. These soluble moieties can be present in the ECM and mediate cell–ECM and cell–cell interactions (33–35).

Thymocyte differentiation and migration occur simultaneously in the thymic microenvironment. T cell progenitors enter the cortico-medullary region via post-capillary venules (36) and rapidly migrate through the cortex toward the subcapsular zone, where DN thymocytes are primarily located. Subsequently, thymocytes migrate to the middle cortex and begin expressing both CD4 and CD8 co-receptors, becoming DP cells. During this stage, cells are selected based on the rearrangement of TCR genes, which leads to the membrane expression of productive TCRs. Cells that do not express productive TCRs undergo apoptosis, whereas cells expressing productive TCRs continue the differentiation process. Then, cells with TCRs that interact with high avidity with MHC-presented self-antigens expressed by mTECs and DCs undergo apoptosis in a process termed negative selection (37). The presentation of self-antigens by mTECs is controlled by Aire and guarantees the deletion of autoreactive T-cell clones, supporting central tolerance (38). In this context, cells with TCRs that interact with low/median avidity with MHC-presented self-antigens survive and continue the maturation process. Survival signals mediated by TCRs and CD4/CD8 co-receptors lead to the down-regulation of a co-receptor, and thymocytes become mature CD4^+^CD8^−^ or CD8^+^CD4^−^ SP cells (Figure 1A).

Thymocyte localization and guidance are controlled by ECM molecules and chemokines, among others molecules, and their respective receptors. For example, the entrance of T-cell progenitors in the thymus is controlled by CCL21/CCR7 and CCL25/CCR9 (chemokine/chemokine receptor) interactions (39). Migration of immature cells within the thymus is controlled by CXCL12/CXCR4 and CCL20/CCR6 interactions (40, 41), and CCR7 signaling is essential for the migration of DP thymocytes to the medulla (42). Moreover, CCR7 is involved in thymocyte egress, which is also controlled by sphingosine-1-phosphate receptor 1 signaling (43). The absence of such molecules in the thymus not only abrogates thymocyte development but also induces changes in the histological organization of the organ (44).

Thymic architecture and organization are essential for proper T-cell development and depend on both the lymphoid and non-lymphoid compartments. Alterations in one compartment can affect the other and consequently modify T-cell development and the repertoire of exported mature T cells to peripheral lymphoid organs. For example, Rag mutations substantially impair thymocyte development and consequently affect the distribution and maturation of TECs, diminishing the proportion of mTECs and inducing a lack of AIRE protein expression (45, 46). Lack of AIRE expression can in turn directly affect negative selection and break central tolerance. Interestingly, Aire deficiency can modulate the intrathymic expression of chemokines as a control mechanism of thymocyte development (47). In this context, one can argue that chemokines and other molecules controlling thymocyte migration (such as ECM molecules) could also modulate Aire expression.

## The Role of Aire in the Ectopic Expression of PTAs in the Thymus

During the induction of central tolerance in the thymus, self-reactive regulatory T cells (T_reg_) are negatively selected, even if these cells play a role in the periphery. In fact, all sets of antigen-presenting cells, including cTECs, mTECs, and thymic DCs, act as self-antigen peptide presenting cells (6–9, 48–53). Thymic DCs present only the PTA peptides that were expressed and processed by mTECs (38).

The expression of PTAs by mTECs is a key process of (auto)immune representation. Due to the wide-ranging diversity of PTAs expressed by these cells, this phenomenon has been termed PGE (5, 9, 12, 48, 54–62).

The primary implication of this type of gene expression, which is heterogeneous and ectopic, is associated with the maintenance of immune homeostasis and controlling the reactivity and self-aggressive autoimmune diseases.

Notably, cTECs and mTECs are essential but not sufficient for these selection events (55). The cTEC-derived signals may regulate the positive selection of thymocytes that recognize the MHC-peptide complexes themselves; however, mTECs that express AIRE help ensure tolerance to self-antigens (63).

A subset of mTECs express the Aire gene (chromosome 10C1 in mice and 21q22.3 in humans) (64) and the claudin proteins (Cld3 or Cld4) on their surface. In these cells, AIRE and the claudin proteins act as adhesion molecules and represent the major proteins that contribute to the molecular architecture of cell junctions. All Cld3^+^, Cld4^+^, and Aire^+^ adult TEC cells strongly express MHC class II and CD80 molecules on their surface (51).

“Immature” CD80^−^/MHC-II^−^ mTECs express a limited set of PTAs, whereas “mature” CD80^+^/MHC-II^+^ mTECs exhibit greater PTA diversity, including PTAs whose expression is Aire dependent (11). These findings have led some researchers to propose “the terminal differentiation model”; i.e., mTECs undergo a continuous process of differentiation similar to the skin or intestinal epithelium, and the full complement of PGE is contingent upon this process (55). mTEC cells are very peculiar due to their unique gene expression pattern. They are capable of expressing more than 19,000 protein-coding genes, including “ectopic” genes that correspond to PTAs. Currently, no other known cell type expresses such a large set of genes (65).

We next sought to determine whether these cells also have unique machinery for gene expression control.

Although the transcriptional control of PGE is partially exerted by Aire, mutations in this gene cause severe autoimmunity that involves various organs and tissues in both mice and humans. In humans, this disease is a syndrome termed APECED, and patients have mutations along the Aire sequence, suggesting that mutations in Aire trigger aggressive autoimmunity (22, 66).

However, the existence of APECED patients who lack Aire mutations (67–69) suggests that other factor(s) may be involved in controlling aggressive autoimmunity. These observations led us to wonder if temporal changes and a slight deregulation of wild-type Aire expression during development could contribute to autoimmunity.

Variation of Aire expression might disturb Aire-dependent PTAs in the thymus and consequently trigger aggressive autoimmunity, a hypothesis that was previously tested by our group (58). This hypothesis is a promising subject for further research and is currently being studied in humans with thymic cells isolated from Down syndrome patients, which feature trisomy of chromosome 21, providing a unique opportunity to evaluate the effect of natural Aire gene dosage in humans (70–72).

The functional role of Aire has been demonstrated using knockout (KO) mice. Aire in mice and humans encodes a protein with affinity for DNA that functions as a positive transcription factor regulating the expression of PTAs mTECs, but AIRE can also act as a negative regulator of other genes (8, 10, 73–76).

The link between Aire expression and the induction of thymocyte apoptosis, a biological process crucial for negative selection, has been demonstrated (37, 77). However, according to our best knowledge, no investigation has assessed this link considering the possible effect of variations in Aire expression in mTECs on adhesion with thymocytes and the induction of apoptosis. Alternatively, Aire-deficient mTEC cells may lose their adhesion ability. This question is still open for further research.

Interestingly, the AIRE targets low-transcribed genes. It interacts with hypomethylated promoter regions in the chromatin through its PHD1 domain (62, 78–82).

However, recent evidence has demonstrated that the AIRE acts indirectly in regulating PTA transcription. According to Giraud et al. (83), AIRE can be considered an unusual transcription factor because it does not appear to function as a typical transactivator. These authors demonstrated that AIRE activates PTA transcription by releasing stalled RNA Pol II from blockage at the promoter region of its target genes. This model suggests that AIRE acts during the elongation stage of transcription rather than at transcription initiation (83). The “promiscuity” of Aire on a large set of downstream PTA genes might be due the unspecific mode of action of RNA Pol II on different promoter regions, but this remains to be determined.

A new exciting possibility for the Aire mechanism is its influence in controlling alternative splicing of PTA genes in mTECs. Aire has been shown to increase the amount of measurable exons per gene and enables the production of PTAs from these exons (84); these properties might significantly increase the diversity of PTA isoforms in mTECs and consequently increase the range of self-representation.

It is possible that aggressive autoimmunity is associated with an imbalance of PTAs isoforms in mTECs.

## The Link between Aire and miRNAs

In our view, not only Aire but also miRNAs may play a role in central tolerance. This hypothesis is plausible considering the vast range of action of miRNAs, which affect more than half of all mRNAs originating from protein-coding genes in human or murine cells (85).

This range of action is expected to reach mRNAs encoding proteins involved in the central tolerance mechanism, including PTA mRNAs and Aire mRNA itself.

First, researchers evaluated the role of the endoribonuclease Dicer, a key enzyme implicated in miRNA maturation, on thymic function. They found that Dicer-KO mice exhibit progressive degeneration in thymic architecture and function, provoking alterations in T cell differentiation and peripheral tolerance, pinpointing miRNA-29a as a specific miRNA participating in this process (29).

Then, mice lacking Dicer expression in the thymic epithelia were found to exhibit a set of abnormalities, including alterations in the expression profiling of cTEC and mTEC mRNAs. T cells obtained from a Dicer-deficient thymus were pathogenic and produced aggressive autoimmunity (86). These finding were instrumental for further research on the role of miRNAs in central tolerance induction.

Moreover, thymic epithelial cells isolated from murine or human thymuses feature overlapping of miRNA signatures, suggesting evolutionary conservation of miRNA expression profiles (87). These authors also demonstrated that Aire expression is associated with maturation-dependent expression of miRNAs.

However, a direct demonstration that Dicer and consequently miRNAs play a role in TEC-thymocyte adhesion, which is crucial for positive and negative selection, is still lacking. This question is open for further investigation.

As discussed above, the AIRE acts in close association with RNA Pol II (83). Because this polymerase transcribes miRNAs in addition to mRNAs (88–92), Aire may affect miRNA expression.

Our group was the first to directly demonstrate this possibility (28). We showed that in murine mTECs, Aire controls the transcription of miRNAs located within a genomic region that encompasses an open-reading frame (ORF of Gm2922 mRNA).

This finding enabled further evaluation of the role played by Aire-dependent miRNAs in the posttranscriptional control of PTAs. Thus, we reconstructed miRNA–mRNA interaction networks from mTECs isolated from BALB/c (non-autoimmune) or non-obese diabetic (NOD) (autoimmune) mice. As expected, dozens of PTA mRNAs interacted with miRNAs. Interestingly, none of the classical Aire-dependent PTAs (e.g., Ins2) interacted with miRNAs, strongly suggesting that they are somewhat resistant to posttranscriptional control (30).

What would be the consequences of a lack of miRNA action on these PTAs? Could this lack of action aid autoantigen synthesis by mTECs, consequently inducing tolerance? What causes these Aire-dependent PTAs to be “resistant” to miRNA action? Could changes in their 3′UTRs (length or mutations) or imbalance in miRNAs expression levels (or both) cause this resistance? We have suggested that there may be changes in length of the 3′UTR sequence of Aire-dependent PTAs expressed in mTECs (30). Researchers including our group and the group of Mathieu Giraud in Paris are now challenged to evaluate the structure of mRNAs in general and/or the 3′UTR of mRNAs of PTAs expressed in mTECs compared with other cell types.

Based on these recent results, is possible to drawn a pathway for the transcriptional and posttranscriptional control of PGE in mTECs (Figure 1B). Within the nuclear compartment, the AIRE controls PTA and miRNA transcription (Aire-dependent PTAs and miRNAs). Once in the cytoplasm, miRNAs play a role in the posttranscriptional control of Aire and PTA mRNAs. Would PTA mRNAs with altered 3′UTRs be refractory to the action of miRNAs?

Although these aspects have only recently begun to be explored, they represent new, exciting questions for present and future research on the molecular genetic basis of immune tolerance.

## Concluding Remarks

The molecular genetic control of central tolerance remains an open question in immunology. The identification and cloning of the Aire gene was instrumental in studying the molecular genetics of this process. As the primary controller of PTA expression in mTECs, Aire is the master pillar of central tolerance. Aire expression is common in the thymus; and this observation led to the idea of PGE. However, Aire did not fit well as a classic transcription factor. The AIRE operates in conjunction with various other partner proteins in the release of RNA Pol II shortly after the initiation of PTA gene transcription. This property enabled better understanding of the vast range of AIRE activity. Recently, miRNAs have been found to be the modulators of post-transcriptional controllers in the thymus. Researchers are now challenged with deciphering the transcriptional and post-transcriptional control pathway of PGE involving Aire and miRNAs.

## Conflict of Interest Statement

The authors declare that the research was conducted in the absence of any commercial or financial relationships that could be construed as a potential conflict of interest.
